# 16S-Pipeline: A comprehensive web-based platform for end-to-end 16S rRNA amplicon sequencing analysis

**DOI:** 10.71150/jm.2603014

**Published:** 2026-05-14

**Authors:** Tatsuya Unno

**Affiliations:** Department of Biological Sciences and Biotechnology, Chungbuk National University, Cheongju 28644, Republic of Korea

**Keywords:** 16S rRNA, amplicon sequencing, microbiome analysis, DADA2, differential abundance, functional prediction, PICRUSt2, bioinformatics pipeline, web application

## Abstract

16S rRNA gene amplicon sequencing is the most widely used approach for characterizing microbial communities, yet analyzing such data requires navigating a fragmented landscape of bioinformatics tools with distinct installation requirements, parameter settings, and data formats. Here we present 16S-Pipeline, an open-source, web-based platform that provides a complete workflow from raw FASTQ files to publication-ready statistical analyses. 16S-Pipeline automatically detects sequencing type (paired-end, single-end, long-read), variable region, and sequencing platform (Illumina, PacBio HiFi, Nanopore), then performs quality filtering, primer trimming, amplicon sequence variant (ASV) inference via DADA2, taxonomy assignment against SILVA v138.1, phylogenetic tree construction, and optional functional prediction via PICRUSt2. Downstream analyses include alpha and beta diversity, taxonomic composition visualization, differential abundance testing using five complementary methods (ALDEx2, DESeq2, ANCOM-BC2, LinDA, MaAsLin2) with consensus reporting, and KEGG pathway mapping. Built-in NCBI SRA integration enables downloading public datasets for re-analysis and generates submission metadata spreadsheets for data deposition. The interactive web interface built on FastAPI and Plotly Dash enables researchers to perform complex microbiome analyses without command-line expertise. 16S-Pipeline is freely available at https://github.com/tatsu1207/16S-Pipeline under the MIT License.

## Overview

Microbial community profiling through 16S rRNA gene amplicon sequencing has become a cornerstone of microbiome research, with applications spanning human health (Rup, 2012), environmental ecology (Thompson et al., 2017), agriculture (Fierer, 2017), and food science (De Filippis et al., 2018). The declining cost of high-throughput sequencing has made 16S amplicon surveys accessible to a broad range of researchers, yet the bioinformatics analysis of the resulting data remains a significant bottleneck.

A standard 16S amplicon-based analysis workflow requires multiple steps: quality assessment of raw reads, primer and adapter removal, sequence denoising or clustering, taxonomic classification, phylogenetic tree construction, and downstream statistical analyses including diversity estimation, community composition visualization, and differential abundance testing. Each step typically relies on a different software tool—FastQC for quality assessment, Cutadapt for primer trimming (Martin, 2011), DADA2 for denoising (Callahan et al., 2016), SILVA for taxonomy (Quast et al., 2013), MAFFT for alignment (Katoh and Standley, 2013), FastTree for phylogeny (Price et al., 2010), and various R packages for statistical analysis. Integrating these tools requires familiarity with command-line environments, R programming, and the specific input/output formats of each tool. This fragmentation creates barriers for researchers who lack dedicated bioinformatics support, particularly in clinical, environmental, and agricultural laboratories.

Several tools and platforms have been developed to address this challenge, each with distinct strengths and limitations (Table 1). DADA2 introduced amplicon sequence variant (ASV) inference, resolving sequences at single-nucleotide resolution rather than clustering at the traditional 97% similarity threshold used by OTU-based approaches. ASVs are directly comparable across studies without re-clustering, improving reproducibility (Callahan et al., 2017). DADA2 also learns sample-specific error rates from the data and provides native support for long-read sequencing platforms through dedicated error models (PacBioErrfun for PacBio HiFi, loessErrfun for Nanopore). However, DADA2 is an R package focused on sequence-level processing — quality filtering, denoising, chimera removal, and taxonomy assignment — and does not provide upstream quality assessment, phylogenetic tree construction, diversity analysis, or differential abundance testing.

MOTHUR (Schloss et al., 2009) offers a comprehensive command-line pipeline covering the full workflow from raw reads to statistical analysis, and has been widely adopted since its introduction. However, MOTHUR relies on OTU clustering at fixed similarity thresholds, which merges biologically distinct sequences and limits taxonomic resolution. As the field has shifted toward ASV-based approaches, MOTHUR's OTU-centric framework has become less aligned with current best practices (Knight et al., 2018). Additionally, MOTHUR requires command-line proficiency and its scripting syntax presents a learning curve for non-bioinformaticians.

QIIME 2 (Bolyen et al., 2019) provides the most comprehensive command-line framework with an extensive plugin architecture. Notably, QIIME 2's most widely used denoising plugin internally wraps DADA2, adding an abstraction layer of artifacts, metadata formats, and plugin management on top of the same core algorithm. While this plugin system offers flexibility, it requires substantial computational literacy for installation and operation, and QIIME 2's conda-based installation is frequently complicated by dependency conflicts. QIIME 2 does not natively include multiple differential abundance methods or functional prediction capabilities without additional plugin installation and configuration.

Web-based alternatives such as Nephele (Weber et al., 2018) and Galaxy-based solutions (Afgan et al., 2018) lower the interface barrier but are hosted externally, raising concerns about data privacy and upload bandwidth for large datasets. MicrobiomeAnalyst (Chong et al., 2020) focuses on downstream statistical analysis but does not process raw sequencing data.

nf-core/ampliseq (Straub et al., 2020) provides a comprehensive Nextflow-based pipeline supporting DADA2 with automated truncation via a quality threshold parameter (--trunc_qmin), but requires familiarity with Nextflow workflow management and command-line operation. IMNGS (Lagkouvardos et al., 2016) offers a web-based interface with integrated SRA data import and OTU-based analysis, but relies on external server processing and uses OTU clustering rather than ASV inference. FIGARO (Weinstein et al., 2019) addresses truncation parameter optimization specifically by modeling expected error rates across parameter combinations, but is a standalone preprocessing tool rather than an end-to-end pipeline. The approach implemented in 16S-Pipeline differs in that it jointly optimizes truncation lengths by maximizing combined read retention (estimated expected errors ≤ 5) while enforcing the overlap constraint that the sum of forward truncation length (trunc_f) and reverse truncation length (trunc_r) must satisfy trunc_f + trunc_r ≥ insert_length + min_overlap, using known amplicon sizes derived from the auto-detected variable region and primer sequences. This region-aware constraint ensures that truncation parameters are always compatible with successful paired-end merging, which is particularly important in an automated pipeline where manual inspection of quality profiles is not expected.

Two additional challenges confront microbiome researchers. First, automated selection of quality filtering parameters—particularly truncation lengths for DADA2—remains largely manual, requiring visual inspection of quality profiles and subjective judgment. Inappropriate truncation lengths can lead to excessive read loss or, in paired-end sequencing, failure of read merging due to insufficient overlap. Second, differential abundance analysis is complicated by the well-documented disagreement among statistical methods (Nearing et al., 2022; Yang and Chen, 2022), making it difficult for researchers to assess the robustness of their findings without running multiple tools and comparing results.

To address these challenges, we developed 16S-Pipeline, an open-source, locally deployable web application that integrates the complete 16S rRNA analysis workflow. Key features include: (1) automated detection of sequencing parameters including type, variable region, and platform with adaptive quality filtering; (2) support for both short-read (Illumina) and long-read (PacBio HiFi, Nanopore) sequencing technologies; (3) multi-method differential abundance analysis with consensus reporting across five established statistical frameworks; (4) integrated functional prediction via PICRUSt2 with interactive KEGG pathway visualization; (5) cross-region dataset merging capabilities; and (6) NCBI SRA integration for downloading public datasets and generating submission metadata.

## Applications

16S-Pipeline is designed for researchers across diverse fields who need to analyze 16S rRNA gene amplicon sequencing data but may lack dedicated bioinformatics support. The platform addresses the needs of microbiome researchers in clinical, environmental, agricultural, and food science laboratories where command-line bioinformatics expertise is not readily available.

Primary applications include: (1) human and animal gut microbiome profiling for health and disease association studies; (2) soil and water environmental microbial community characterization, such as studies involving microbial source tracking (Song and Unno, 2024); (3) food fermentation and safety monitoring; (4) agricultural microbiome analysis for crop and livestock improvement; and (5) any study requiring 16S rRNA-based microbial community profiling with publication-ready statistical output.

The web-based interface makes the platform particularly suited for core facilities serving multiple research groups, teaching laboratories introducing students to microbiome analysis, and individual researchers who need to perform complex analyses without programming expertise. Support for Illumina short-read, PacBio HiFi, and Oxford Nanopore long-read sequencing within a unified interface accommodates laboratories using different sequencing platforms. Local deployment ensures data privacy, which is critical for clinical and veterinary studies subject to data protection regulations.

## Methods

### Architecture

**Fig. 1.**
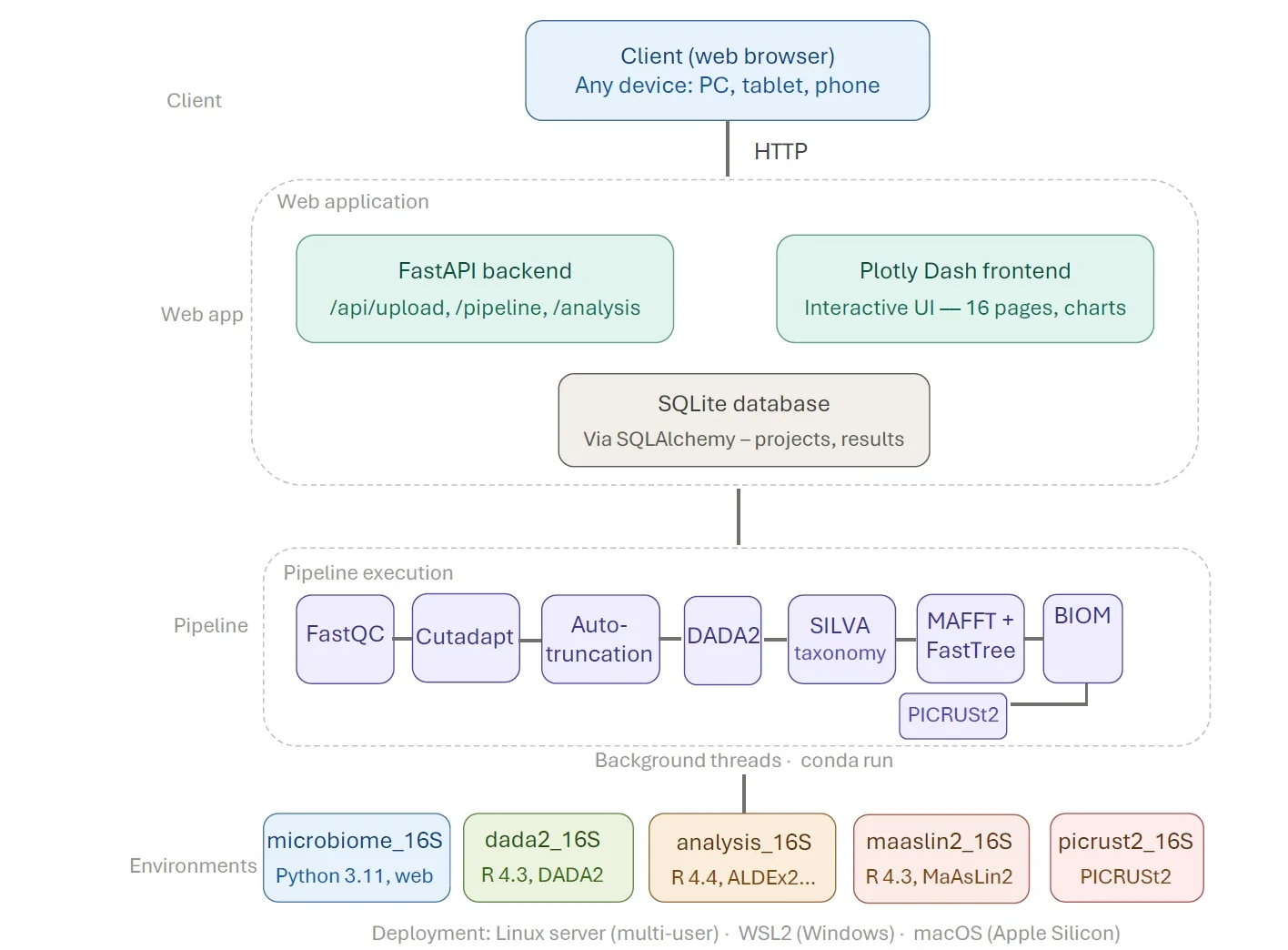
Architecture overview of 16S-Pipeline. The platform consists of a FastAPI backend serving RESTful API endpoints, a Plotly Dash frontend for interactive visualization, an SQLite database for metadata and result storage, and five isolated conda environments for pipeline execution and statistical analysis. R scripts are executed via subprocess calls for DADA2 denoising, taxonomy assignment, and differential abundance testing.

16S-Pipeline is implemented as a client-server web application (Fig. 1). The backend is built on FastAPI, providing RESTful API endpoints for data upload, pipeline management, and analysis execution. The interactive frontend is implemented using Plotly Dash with Bootstrap styling, mounted as a sub-application within the FastAPI server. Data persistence is handled by SQLite via SQLAlchemy 2.0, storing project metadata, upload records, sample information, and cached analysis results across 12 relational tables.

The application employs five isolated conda environments to manage complex dependency requirements (Table S1). This multi-environment architecture ensures reproducible dependency management while avoiding version conflicts between R packages—notably, MaAsLin2 requires R 4.3 while newer versions of ALDEx2 and ANCOM-BC2 benefit from R 4.4, necessitating separate environments. R scripts are executed via subprocess calls using conda run, with stdout/stderr streaming to the web interface for real-time progress monitoring.

### Considerations and limitations

Several design decisions distinguish 16S-Pipeline from existing tools. The automated quality parameter selection eliminates a common source of irreproducibility in amplicon analyses, while the integration of five differential abundance methods with consensus reporting aligns with recent recommendations to assess result robustness across multiple statistical frameworks (Nearing et al., 2022). Support for both short-read and long-read sequencing within a unified interface positions the platform for the growing adoption of full-length 16S sequencing technologies, which enable species-level taxonomic resolution that short-read approaches targeting individual variable regions cannot reliably achieve (Callahan et al., 2019). Local deployment ensures data privacy, which is particularly important for clinical and veterinary microbiome studies.

The platform has certain limitations. While 16S-Pipeline supports Linux, Windows (via WSL2), and macOS (Apple Silicon), the macOS deployment relies on Rosetta 2 emulation for PICRUSt2 due to the lack of native ARM64 builds. The platform focuses on amplicon-based analyses and does not support shotgun metagenomic data. Species-level taxonomic resolution from short-read data is inherently limited by the partial 16S marker gene coverage, though full-length 16S long-read sequencing can improve species identification. Functional predictions via PICRUSt2 are inferential rather than directly measured.

When merging datasets across studies or sequencing runs, batch effects due to differences in DNA extraction, PCR amplification, library preparation, or sequencing platform may confound biological signals. 16S-Pipeline provides two merging modes — by-sequence for same-region datasets and by-taxonomy for cross-region datasets — but does not currently implement computational batch correction methods such as ComBat (Johnson et al., 2007) or MMUPHin (Ma et al., 2022). Users should interpret cross-study comparisons cautiously and consider including study or batch as a covariate in downstream statistical analyses. The platform's metadata framework supports such covariates in the differential abundance tools that accept multivariable models (e.g., MaAsLin2).

Dependency management and reproducibility are addressed through several mechanisms. Each of the five conda environments specifies tool versions at installation time via conda’s dependency resolver, and the setup script pins major versions (e.g., R 4.3 for DADA2, R 4.4 for AlDEx2/ANCOM-BC2) to prevent breaking changes from upstream updates. The Docker deployment provides fully reproducible environments, as each release produces a self-contained image with all dependencies frozen at build time. Docker image tags follow semantic versioning, allowing users to pin to a specific release. For native installations, the setup_ubuntu.sh script produces consistent environments by specifying channel priorities and package versions. The SILVA v138.1 reference database is downloaded at installation time and remains fixed unless the user explicitly updates it. The platform's GitHub repository uses tagged releases, and the DADA2 parameter table (Table S3) documents all algorithm settings to support methodological reproducibility. We acknowledge that long-term maintenance of five conda environments with R/Python cross-dependencies requires ongoing attention, and we are committed to maintaining compatibility through regular testing and versioned releases.

## Materials

16S-Pipeline supports two deployment modes. In the server deployment mode, the application is installed on a shared Linux server (Ubuntu 20.04+) using setup_ubuntu.sh, which automates the installation of all five conda environments, Python and R dependencies, and the SILVA v138.1 reference database. Multiple users access the platform through their web browsers, each running an independent instance on a user-specific port (7000 + user UID). In the local deployment mode, a Docker image provides a fully self-contained installation for Windows, macOS, and Linux, requiring no manual dependency management. The Docker deployment is the recommended method for individual workstations, as it eliminates platform-specific compatibility issues and ensures reproducible environments across operating systems. The Docker image supports both AMD64 and ARM64 architectures. However, PICRUSt2 is not available in the ARM64 build (including macOS with Apple Silicon) due to the absence of a native ARM64 package in the Bioconda repository. The platform handles this gracefully — all other pipeline and analysis features remain fully functional, and the PICRUSt2 page displays a notification when the tool is unavailable.

A step-by-step tutorial using publicly available soil microbiome data (BioProject PRJNA1192699) is provided in the repository (TUTORIAL.md), guiding users through the complete workflow from data download to publication-ready results. Subsampled test datasets (5,000 reads per sample) are also included for rapid evaluation of all platform features.

## Protocols

### Pipeline workflow

**Fig. 2.**
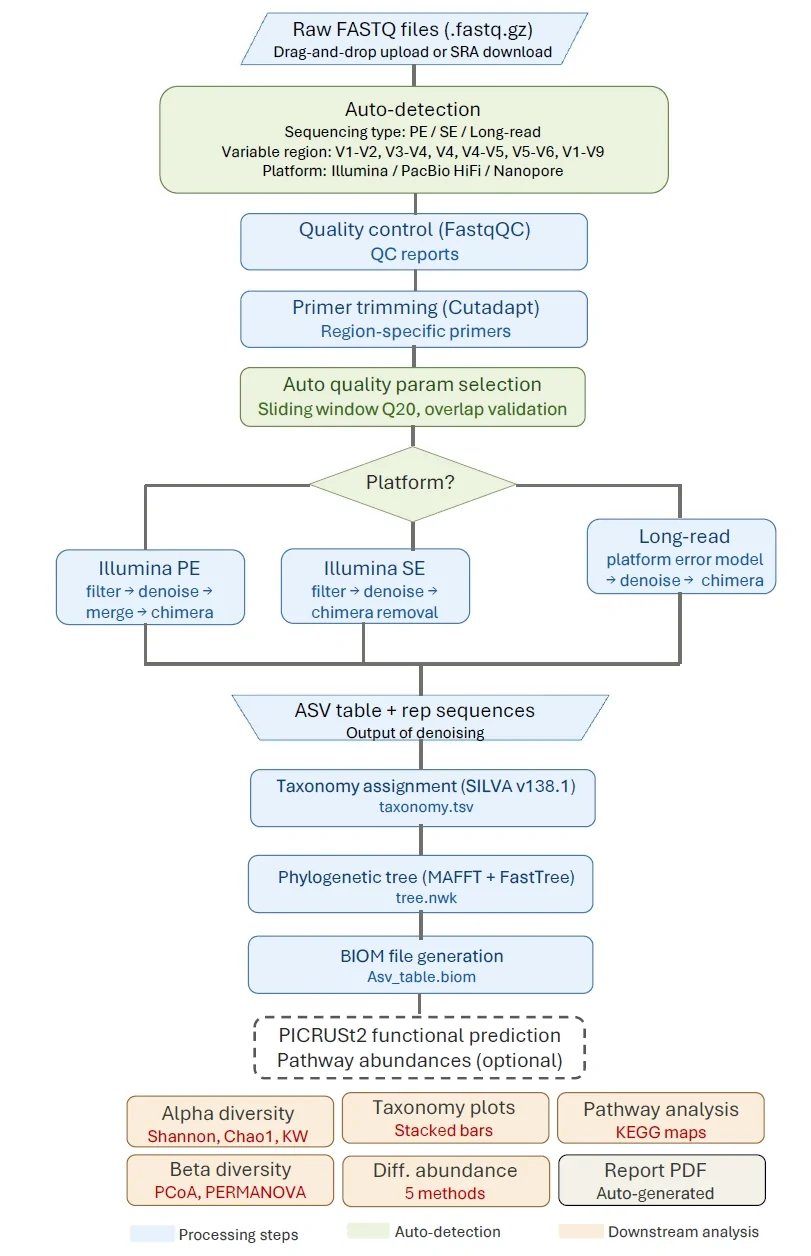
16S-Pipeline processing workflow. Raw FASTQ files are uploaded through the web interface, where sequencing type, variable region, and platform are automatically detected. The pipeline proceeds through quality control (FastQC), primer trimming (Cutadapt), automated quality parameter selection, ASV inference (DADA2), taxonomy assignment (SILVA v138.1), phylogenetic tree construction (MAFFT + FastTree), BIOM file generation, and optional functional prediction (PICRUSt2). Downstream analyses include alpha and beta diversity, taxonomic composition, multi-method differential abundance testing, and KEGG pathway analysis.

The 16S-Pipeline workflow consists of up to eight sequential stages, each executed in a background thread with real-time progress tracking through the web interface (Fig. 2).

**Data upload and automatic detection.** Users upload compressed FASTQ files (.fastq.gz) through a chunked drag-and-drop interface capable of handling large files without browser blocking. Upon upload, 16S-Pipeline automatically determines: (i) sequencing type (paired-end vs. single-end) from filename conventions with file pairing validation; (ii) variable region through regex-based matching of known primer sequences with full IUPAC ambiguity support, with detection requiring presence in >30% of sampled reads; and (iii) sequencing platform, with long-read sequencing identified when the average read length exceeds 900 bp. Users can attach sample metadata in CSV or TSV format for use in downstream statistical analyses.

**Quality control and primer trimming.** Quality assessment is performed using FastQC (Andrews, 2010), generating per-sample quality reports accessible through the web interface. Region-specific primer sequences are removed using Cutadapt (Martin, 2011) with anchored 5' matching. For paired-end data, forward and reverse primers are trimmed coordinately. For long-read data, bidirectional trimming accommodates reads in both orientations.

**Automated quality parameter selection.** A critical feature of 16S-Pipeline is the automated detection of truncation parameters, implemented as a pipeline-specific heuristic that approximates the manual quality inspection recommended in standard DADA2 workflows (Callahan et al., 2016). The algorithm samples 5,000 reads evenly distributed across each FASTQ file to avoid sequencing-run position bias, computes per-base mean Phred quality scores, and identifies the truncation point as the rightmost position where a 10 bp sliding window maintains mean quality ≥ Q20. For paired-end data, the algorithm jointly optimizes forward and reverse truncation lengths by enforcing the overlap constraint trunc_f + trunc_r ≥ insert_length + min_overlap, where insert length is derived from the known amplicon size of the detected variable region. Among truncation pairs satisfying this constraint, the algorithm selects the combination maximizing combined read retention, estimated as the product of per-direction pass rates at expected errors ≤ 5. Forward reads are extended preferentially when additional overlap is needed, as they typically maintain higher quality. Truncation lengths are additionally capped at the insert length to prevent sequencing into adapter. Users retain the option to override auto-detected parameters through the web interface.

**ASV inference via DADA2.** Amplicon sequence variant inference is performed using DADA2 (Callahan et al., 2016), supporting three processing modes: paired-end Illumina (quality filtering, denoising, read merging, and chimera removal), single-end Illumina (quality filtering, denoising, and chimera removal), and long-read PacBio HiFi/Nanopore (platform-specific error models, adjusted banding parameters, homopolymer gap penalties, and a length filter of 1,000–1,600 bp). Outputs include an ASV count table, representative sequences in FASTA format, and per-sample read tracking statistics. The complete DADA2 parameter settings used by 16S-Pipeline are summarized in Table S3. For Illumina short-read data, filterAndTrim() is called with maxN=0, maxEE=c(5,5) for paired-end or maxEE=5 for single-end, truncQ=0 (disabled, as truncation is handled by the auto-detection step), and rm.phix=TRUE. The truncLen parameter is either auto-detected (default) or user-specified through the web interface. Error rates are learned via learnErrors() with default parameters (loessErrfun, randomize=TRUE). Denoising uses dada() with sample-independent processing by default (pool=FALSE), with pseudo-pooling (pool="pseudo") available as a user option. For paired-end data, reads are merged with mergePairs() using minOverlap=12 (user-adjustable) and maxMismatch=1. Chimeras are removed with removeBimeraDenovo() using the "consensus" method.

For long-read data (PacBio HiFi, Oxford Nanopore), the pipeline uses platform-specific error models: PacBioErrfun for PacBio and loessErrfun for Nanopore. Additional long-read parameters include BAND_SIZE=32, HOMOPOLYMER_GAP_PENALTY=-1, length filtering (minLen=1000, maxLen=1600), and maxEE=10. These follow the recommendations of Callahan et al. (2019) for full-length 16S analysis.

**Taxonomy assignment.** Taxonomy is assigned using DADA2's naive Bayesian classifier (Wang et al., 2007) against the SILVA NR99 v138.1 reference database (Quast et al., 2013). Genus-level classification uses assignTaxonomy(), followed by exact-match species-level assignment via addSpecies(). Full-length 16S sequences generated by long-read platforms (PacBio HiFi, Nanopore) provide substantially higher species-level resolution than short-read data targeting individual variable regions, as the ~1,500 bp sequences are more likely to yield unique exact matches against the SILVA species reference database.

**Phylogenetic tree construction.** Representative ASV sequences are aligned using MAFFT (Katoh and Standley, 2013) and an approximate maximum-likelihood tree is constructed using FastTree 2 (Price et al., 2010) under the GTR+CAT model. The resulting tree is stored for potential use in phylogeny-aware analyses.

**BIOM file generation.** The ASV count table and taxonomy assignments are combined into a single HDF5-format BIOM file (McDonald et al., 2012) with representative sequences embedded as observation metadata.

**Functional prediction (optional).** PICRUSt2 (Douglas et al., 2020) functional prediction is optionally executed in a dedicated conda environment. Due to its substantial memory requirements (> 11 GB RAM), this step can fail independently without affecting the core pipeline results.

### Data management

16S-Pipeline provides several data management capabilities between pipeline processing and statistical analysis. Users can rarefy datasets to uniform sequencing depth (Weiss et al., 2017), with the interface displaying per-sample read counts and allowing exclusion of low-depth samples before rarefaction. ASVs can be filtered based on minimum prevalence and minimum relative abundance thresholds to reduce noise from potential sequencing artifacts. Rarefaction depth is user-defined, with the interface providing data-driven guidance: per-sample read counts are displayed and the minimum sample depth is pre-filled as the default rarefaction target. An interactive threshold tool allows users to preview the number of samples retained at any depth, facilitating the trade-off between depth and sample inclusion before rarefaction is applied.

The platform supports merging multiple processed datasets for meta-analyses across studies or sequencing runs. Same-region datasets are merged by sequence identity, while cross-region datasets are harmonized by taxonomy using *Escherichia coli* 16S rRNA alignment positions to identify corresponding V-region boundaries. BIOM files can also be converted to MOTHUR-compatible shared and taxonomy file formats.

### NCBI SRA integration

16S-Pipeline provides built-in integration with the NCBI Sequence Read Archive (SRA) to streamline both data acquisition and data sharing.

**SRA downloader.** Users can download public 16S rRNA datasets directly within the platform by entering SRR (run), SRP (study), or PRJNA (BioProject) accession numbers. The platform resolves study-level accessions to individual run accessions via the NCBI E-utilities API, then downloads and converts SRA files to FASTQ format using prefetch and fasterq-dump from the SRA Toolkit (Leinonen et al., 2011). Downloads are parallelized with up to three concurrent workers. A two-level fallback strategy handles download failures: prefetch is attempted first for SRA file retrieval, falling back to direct download if unsuccessful; fasterq-dump is used for FASTQ conversion, with fastq-dump as a fallback for formats such as PacBio that require it. File integrity is validated by the SRA Toolkit's built-in checksum verification during prefetch, and read counts are computed during registration as an additional sanity check. NCBI API keys are supported through the standard SRA Toolkit configuration. Downloaded files are automatically registered with sequencing type, variable region, and platform detection, making them immediately available for pipeline analysis. There is no hard limit on the number of accessions per job, though the interface warns against mixing multiple project-level accessions that may use different sequencing platforms.

**SRA submission helper.** To support data sharing, 16S-Pipeline generates NCBI SRA submission metadata spreadsheets from existing upload records. The platform auto-populates required SRA fields (library_strategy, library_source, library_selection, library_layout, platform, filetype, filenames) from the detected sequencing parameters, and provides an interface for users to fill in study-specific fields (BioProject accession, instrument model, title, design description). The resulting TSV file conforms to the NCBI SRA submission template, reducing the barrier to depositing raw sequencing data in public repositories.

### Statistical analysis

All statistical analyses are performed through interactive web pages with real-time parameter adjustment and publication-quality figure export (PNG, SVG, PDF).

**Alpha diversity** is computed using multiple metrics: Shannon entropy, Simpson index, inverse Simpson, observed ASV richness, Chao1 estimator, and ACE estimator. Results are visualized as box plots grouped by user-selected metadata categories, with statistical significance assessed using Kruskal-Wallis tests and Dunn’s post-hoc pairwise comparisons.

**Beta diversity** distances are computed using Bray-Curtis dissimilarity and Jaccard distance. Community structure is visualized through Principal Coordinates Analysis (PCoA) and Non-Metric Multidimensional Scaling (NMDS), implemented via R’s vegan package. Statistical assessment of community differences between groups is performed using PERMANOVA (adonis2) with 999 permutations.

**Taxonomic composition** is displayed as interactive stacked bar plots at all taxonomic ranks (Phylum through Species), with user-selectable numbers of top taxa and remaining taxa collapsed into an "Others" category.

**Fig. 3.**
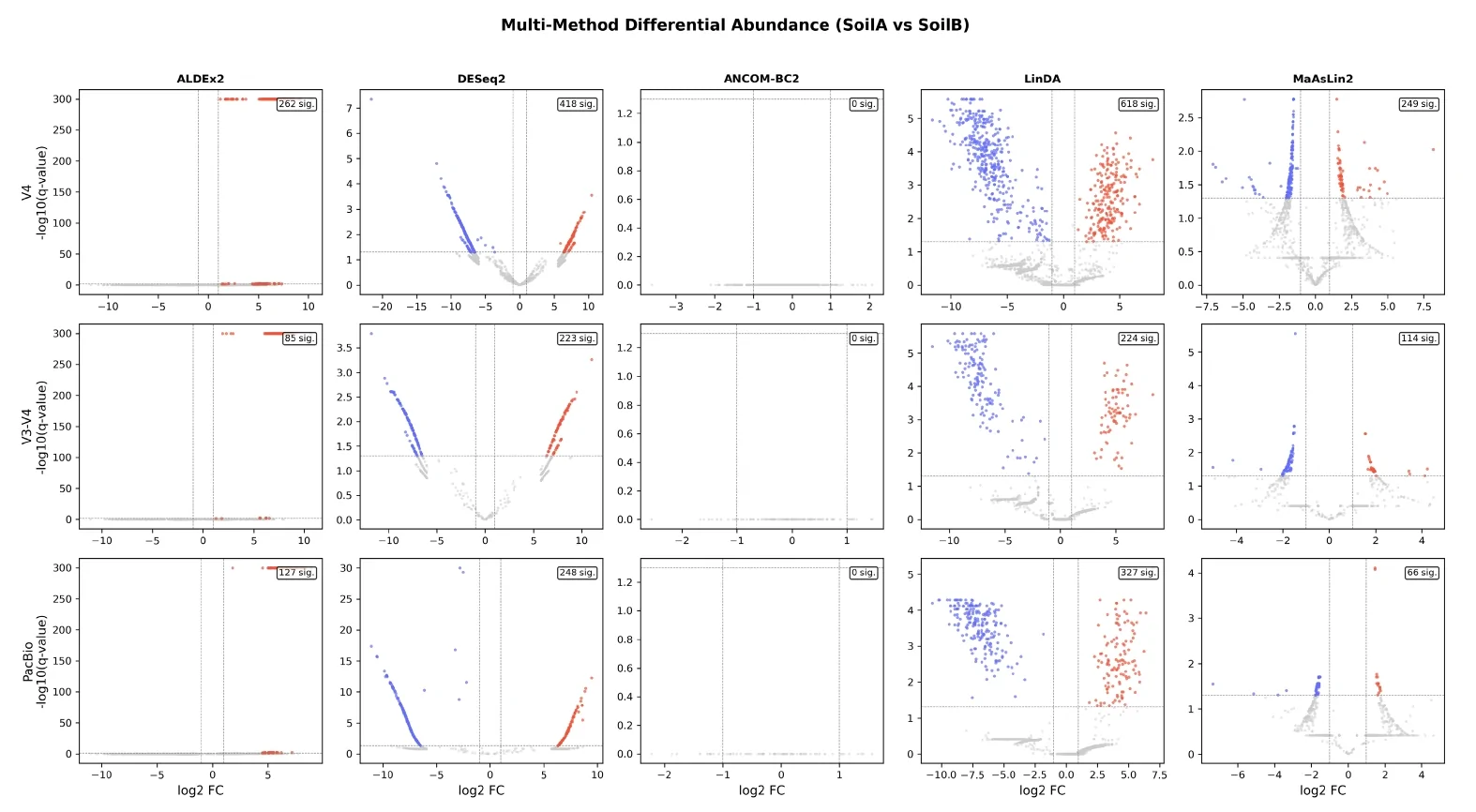
Multi-method differential abundance analysis of the V3-V4 Veselovsky dataset (SoilA vs. SoilB). Volcano plots generated by each of the five statistical methods (ALDEx2, DESeq2, ANCOM-BC2, LinDA, MaAsLin2). The number of significant ASVs (*q* < 0.05) varied from 0 (ANCOM-BC2) to 224 (LinDA), illustrating the importance of multi-method consensus. A total of 148 ASVs were identified as significant by three or more methods.

**Differential abundance analysis.** A distinguishing feature of 16S-Pipeline is the integration of five established differential abundance methods (Fig. 3), addressing the well-documented challenge that different methods can yield substantially different results on the same dataset (Nearing et al., 2022): ALDEx2 (Fernandes et al., 2014), DESeq2 (Love et al., 2014), ANCOM-BC2 (Lin and Peddada, 2020), LinDA (Zhou et al., 2022), and MaAsLin2 (Mallick et al., 2021). Results are presented in a unified comparison table with volcano plots for each tool. A consensus summary highlights ASVs detected by multiple methods, providing researchers with a robust assessment of differential abundance.

**Functional prediction analysis.** When PICRUSt2 results are available, the same five-method comparison framework is applied to predicted MetaCyc pathway abundances. An interactive KEGG pathway viewer (Fig. S1) aggregates predicted KO and EC numbers into KEGG pathway maps, with visualization options including heatmaps, error bar plots, and PCA ordination of functional profiles.

**Outlier detection.** An outlier detection module identifies samples with atypical community compositions based on beta diversity distances, helping researchers identify technical artifacts or biological anomalies before downstream analysis.

**Analysis report generation.** 16S-Pipeline can generate comprehensive PDF reports summarizing all key analyses for a dataset, including dataset summary statistics, an auto-generated Materials & Methods paragraph, alpha and beta diversity plots with statistical test results, taxonomy composition at multiple ranks (Phylum, Family, Genus), and read tracking through pipeline stages. This feature is designed for researchers who need to quickly compile results for lab meetings, collaborator reports, or manuscript preparation.

## Expected Results

To demonstrate that 16S-Pipeline produces correct, biologically meaningful results across supported sequencing platforms, we processed three datasets from the same soil microbiome study (Veselovsky et al., 2025) using different 16S rRNA amplicon strategies: V4 Illumina paired-end, V3-V4 Illumina paired-end, and full-length (V1-V9) PacBio HiFi sequencing. Each dataset contains six samples from two soil types (SoilA and SoilB, n = 3 per group), enabling cross-platform comparison of community profiling results. Raw sequencing data were downloaded from NCBI SRA using 16S-Pipeline's built-in SRA downloader (BioProject PRJNA1192699).

### Pipeline processing

All three datasets were processed through the automated pipeline with default parameters (Table 2). 16S-Pipeline correctly auto-detected the sequencing type, variable region, and platform for each dataset without user intervention. For the V4 dataset, auto-truncation selected forward and reverse truncation lengths of 231 bp and 121 bp, yielding 131 bp of overlap and 49.1% overall read retention (224,747 of 457,861 reads). The V3-V4 dataset required longer truncation lengths (234 bp forward, 230 bp reverse) due to the larger amplicon size (~420–480 bp), resulting in a tighter 22 bp overlap and 29.7% retention—expected for V3-V4 amplicons with 300 bp paired-end reads. The PacBio dataset was processed in long-read mode with platform-specific error models (PacBioErrfun), retaining 48.3% of reads (115,205 of 238,667) after length filtering (1,000–1,600 bp) and chimera removal. The three datasets yielded 5,413, 2,079, and 5,284 ASVs, respectively.

### Taxonomic classification and species-level resolution

Taxonomy was assigned using the SILVA NR99 v138.1 database for all three datasets (Table S2). At higher taxonomic ranks (Kingdom through Class), classification rates were consistently high across platforms (≥ 98%). However, classification rates diverged at lower ranks. At the genus level, the full-length PacBio data achieved 51.1% classification, compared to 47.6% for V3-V4 and 41.4% for V4 Illumina data. Most notably, species-level classification was achieved only with PacBio full-length sequences (0.8%, 41 ASVs), while both short-read datasets yielded 0% species-level assignment. This demonstrates the advantage of full-length 16S sequencing for achieving finer taxonomic resolution.

**Fig. 4.**
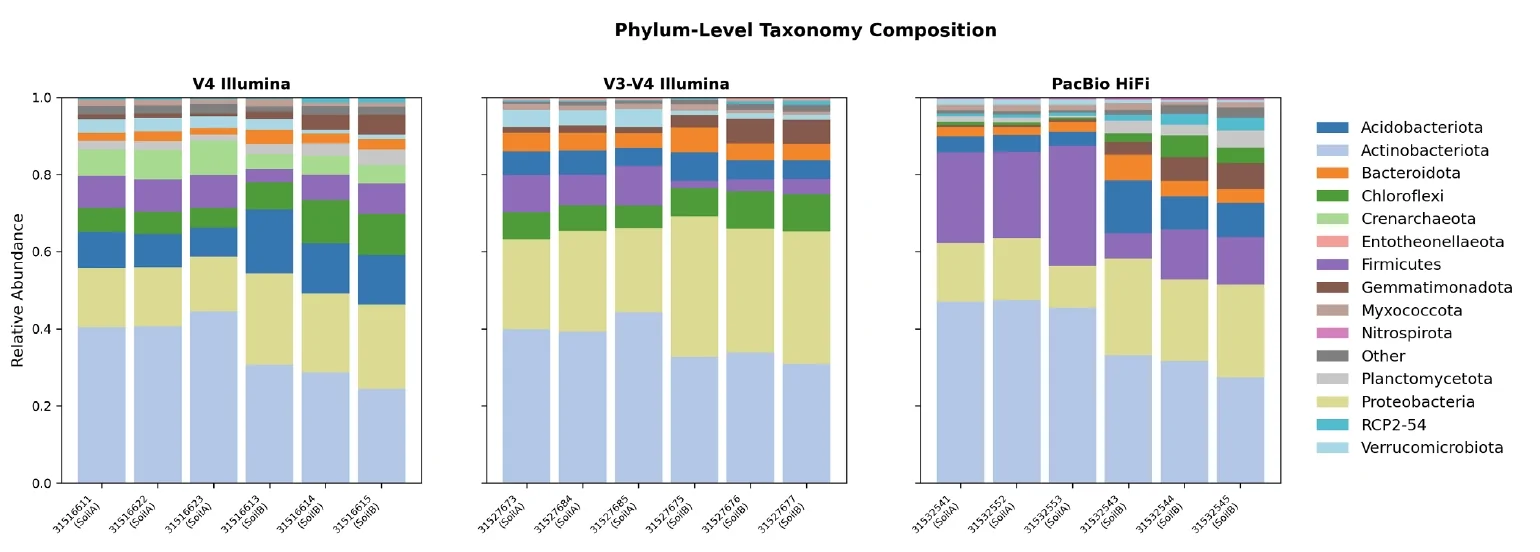
Phylum-level taxonomic composition across three sequencing platforms. Stacked bar plots show relative abundance of the top 15 phyla for each sample across the V4 Illumina (left), V3-V4 Illumina (center), and PacBio HiFi (right) datasets. Consistent dominance of Actinobacteriota, Proteobacteria, and Firmicutes is observed across platforms, validating cross-platform taxonomy assignment.

Community composition was consistent across platforms at the phylum level (Fig. 4). Actinobacteriota was the dominant phylum in all three datasets (35.2–38.8%), followed by Proteobacteria (18.4–29.0%), Firmicutes (6.1–18.1%), and Acidobacteriota (5.7–11.3%)—a profile typical of temperate soil microbiomes. The PacBio dataset showed a notably higher relative abundance of Firmicutes (18.1% vs. 6–7% for Illumina), likely reflecting improved classification of Firmicutes taxa that are ambiguous when only partial 16S sequences are available.

### Alpha and beta diversity

**Fig. 5.**
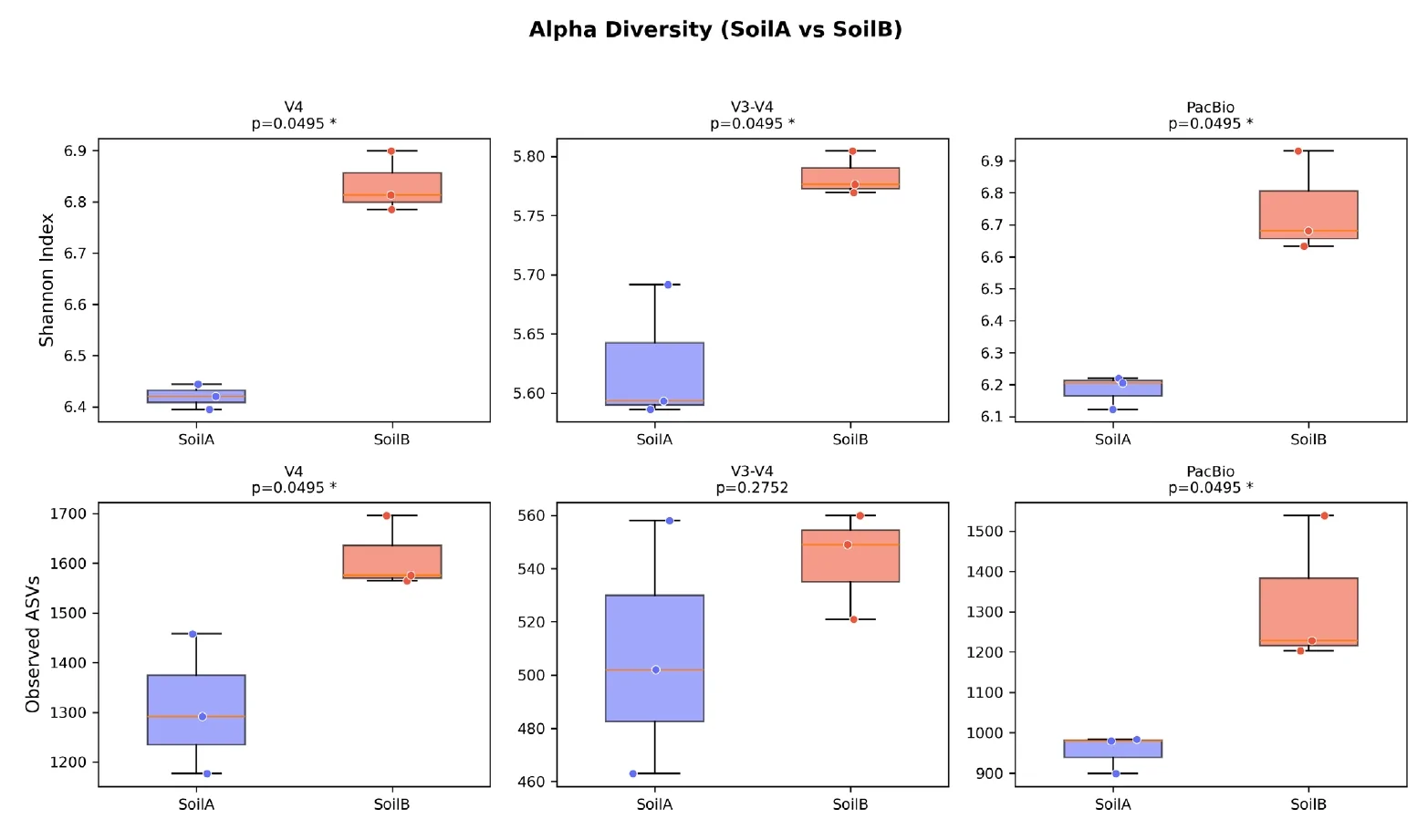
Alpha diversity comparison across three sequencing platforms. Box plots of Shannon diversity index for SoilA and SoilB groups in each dataset. SoilB showed significantly higher Shannon diversity in all three datasets (Kruskal-Wallis, *p* = 0.0495), demonstrating consistent detection of biological differences regardless of sequencing platform.

Alpha diversity analysis detected consistent biological differences between soil types across all three platforms (Fig. 5). Shannon diversity was significantly higher in SoilB than SoilA for all datasets (Kruskal-Wallis, *p* = 0.0495 for all three). Observed ASV richness showed the same trend, reaching significance for V4 (1,309 vs. 1,612, *p* = 0.0495) and PacBio (954 vs. 1,324, *p* = 0.0495) but not for V3-V4 (508 vs. 543, *p* = 0.275), likely due to the lower sequencing depth after quality filtering in the V3-V4 dataset.

**Fig. 6.**
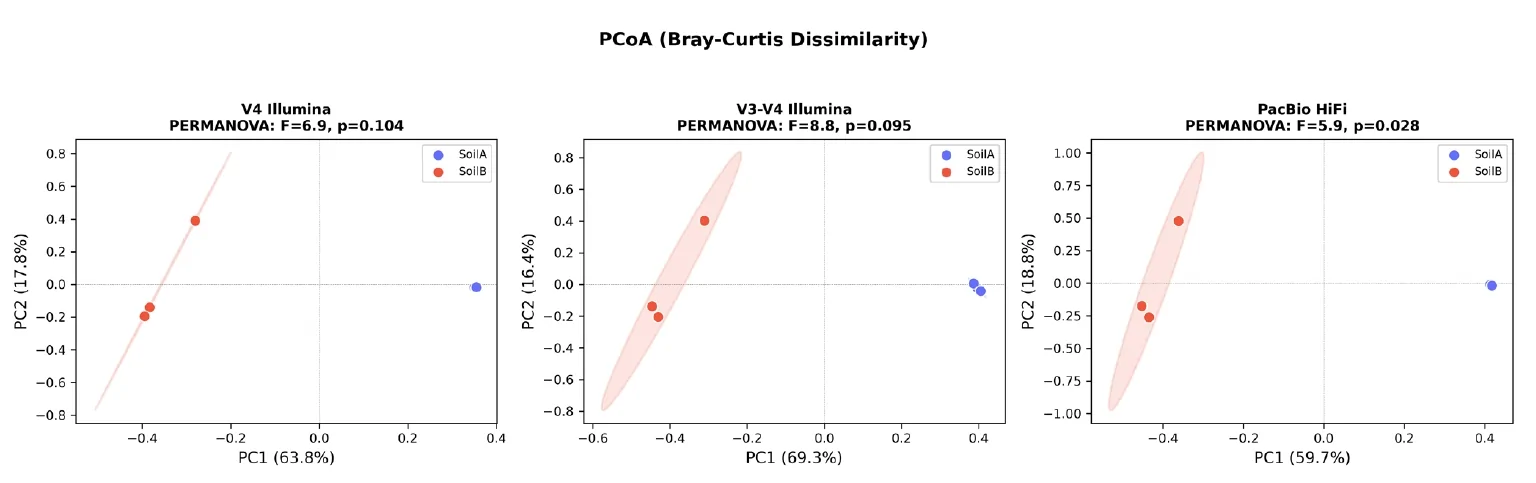
Beta diversity analysis across three sequencing platforms. PCoA ordination based on Bray-Curtis dissimilarity for each dataset, with 95% confidence ellipses drawn for each soil type group. Clear separation of SoilA and SoilB is observed along PC1 (59.7–69.3% variance explained) in all three datasets, confirming that the biological signal is robust across sequencing platforms.

PCoA based on Bray-Curtis dissimilarity revealed clear separation of SoilA and SoilB samples along the first principal coordinate in all three datasets (Fig. 6), with PC1 explaining 59.7–69.3% of the variation. SoilA samples clustered tightly in all datasets, while SoilB samples showed greater dispersion. PERMANOVA tests yielded strong pseudo-F statistics (5.9–8.8) across all platforms, with *p*-values of 0.09–0.11. The marginal significance reflects the limited permutation space with n = 3 per group (maximum C(6,3) = 20 unique permutations), rather than weak biological signal, as the effect sizes are large.

### Multi-method differential abundance analysis

To demonstrate the multi-method consensus feature, we performed differential abundance analysis on the V3-V4 dataset using all five integrated methods (Fig. 3). The number of significantly differentially abundant ASVs (*q* < 0.05) varied considerably across methods: ALDEx2 identified 84, DESeq2 223, ANCOM-BC2 0, LinDA 224, and MaAsLin2 114. This variation exemplifies why reliance on a single method can be misleading, as highlighted by Nearing et al. (2022). ANCOM-BC2 detected no significant features, consistent with its known conservatism at small sample sizes. The consensus analysis identified 148 ASVs as significant by three or more methods, with over 20 ASVs significant by four of five methods, providing a robust set of differentially abundant taxa between the two soil types.

## Future Directions

16S-Pipeline addresses a critical gap in the microbiome analysis tool landscape by providing a locally deployable, web-based platform that covers the entire workflow from raw sequencing data to multi-method statistical analysis. By building on DADA2's ASV inference algorithm while providing a complete end-to-end workflow through a browser interface, 16S-Pipeline combines the analytical rigor of established bioinformatics tools with accessibility for researchers without computational expertise.

Future development priorities include integration of additional diversity metrics and support for ITS amplicon sequencing for fungal community profiling. As microbiome research continues to evolve (Jung, 2025), accessible analysis platforms such as 16S-Pipeline will play an increasingly important role in enabling rigorous and reproducible community profiling.

## Figures and Tables

**Table 1.** Comparison of major 16S rRNA analysis tools

Feature	DADA2	MOTHUR	QIIME 2	nf-core/ampliseq	IMNGS	16S-Pipeline
Sequence resolution	ASV	OTU (97%)	ASV (via DADA2)	ASV (via DADA2)	OTU (97%)	ASV
User interface	R scripting	Command-line	Command-line	Command-line (Nextflow)	Web browser	Web browser
Coding required	Yes (R)	Yes	Yes	Yes (Nextflow)	No	No
End-to-end pipeline	No	Yes	Yes	Yes	Yes	Yes
Auto parameter detection	No	No	No	Yes (--trunc_qmin)	No	Yes
Long-read support	Yes (manual)	Limited	Via plugins	Yes	No	Yes (auto)
DA methods built-in	No	Limited	ANCOM only	Limited (ANCOM)	No	5 methods + consensus
Functional prediction	No	No	Via plugin	Via plugin	No	PICRUSt2 + KEGG
SRA integration	No	No	No	No	Yes (import)	Download + submit
Installation	R package	Binary	conda (complex)	conda/Docker (complex)	External server	Single script

**Table 2.** Pipeline processing summary for three Veselovsky et al. (2025) soil microbiome datasets

Dataset	Region	Seq. type	Samples	Input reads	Output reads	Retention (%)	ASVs	Auto-detected truncation
V4 Illumina	V4	PE 300 bp	6	457,861	224,747	49.1	5,413	F = 231, R = 121 (overlap = 131 bp)
V3-V4 Illumina	V3-V4	PE 300 bp	6	288,177	85,542	29.7	2,079	F = 234, R = 230 (overlap = 22 bp)
PacBio HiFi	V1-V9	Long-read	6	238,667	115,205	48.3	5,284	Long-read mode (1,000–1,600 bp)

## Data Availability

16S-Pipeline source code is available at https://github.com/tatsu1207/16S-Pipeline under the MIT License. The validation datasets used in this study are publicly available from NCBI SRA under BioProject PRJNA1192699 (Veselovsky et al., 2025).

## Supplementary Information

Table S1. Conda environments used by 16S-Pipeline

Table S2. Percentage of ASVs classified at each taxonomic rank across three sequencing platforms

Table S3. DADA2 parameter settings used by 16S-Pipeline across Illumina paired-end, Illumina single-end, and long-read (PacBio HiFi/Nanopore) processing modes, indicating whether each parameter is fixed, automatically detected, or user-adjustable

Fig. S1. PICRUSt2 functional prediction and KEGG pathway analysis. (A) Pathway Analysis page showing MetaCyc pathway comparison between SoilA and SoilB with error bar plots and differential abundance results. (B) KEGG Pathway Map page for Oxidative phosphorylation (map00190), displaying KO coverage (106/224 KOs detected), pathway activity box plot comparing groups, and per-KO abundance table with statistical results. (C) KEGG pathway map with detected enzymes colored by differential abundance between groups.
