# Supplementary material for: 16S-Pipeline: A comprehensive web-based platform for end-to-end 16S rRNA amplicon sequencing analysis
Source: J Microbiol. 2026 May 14;64(5):e2603014. doi: 10.71150/jm.2603014 (PMC13577063; doi:10.71150/jm.2603014)
Supplement: Supplementary file 1 [file jm-2603014-Supplementary-Fig-S1.pdf]

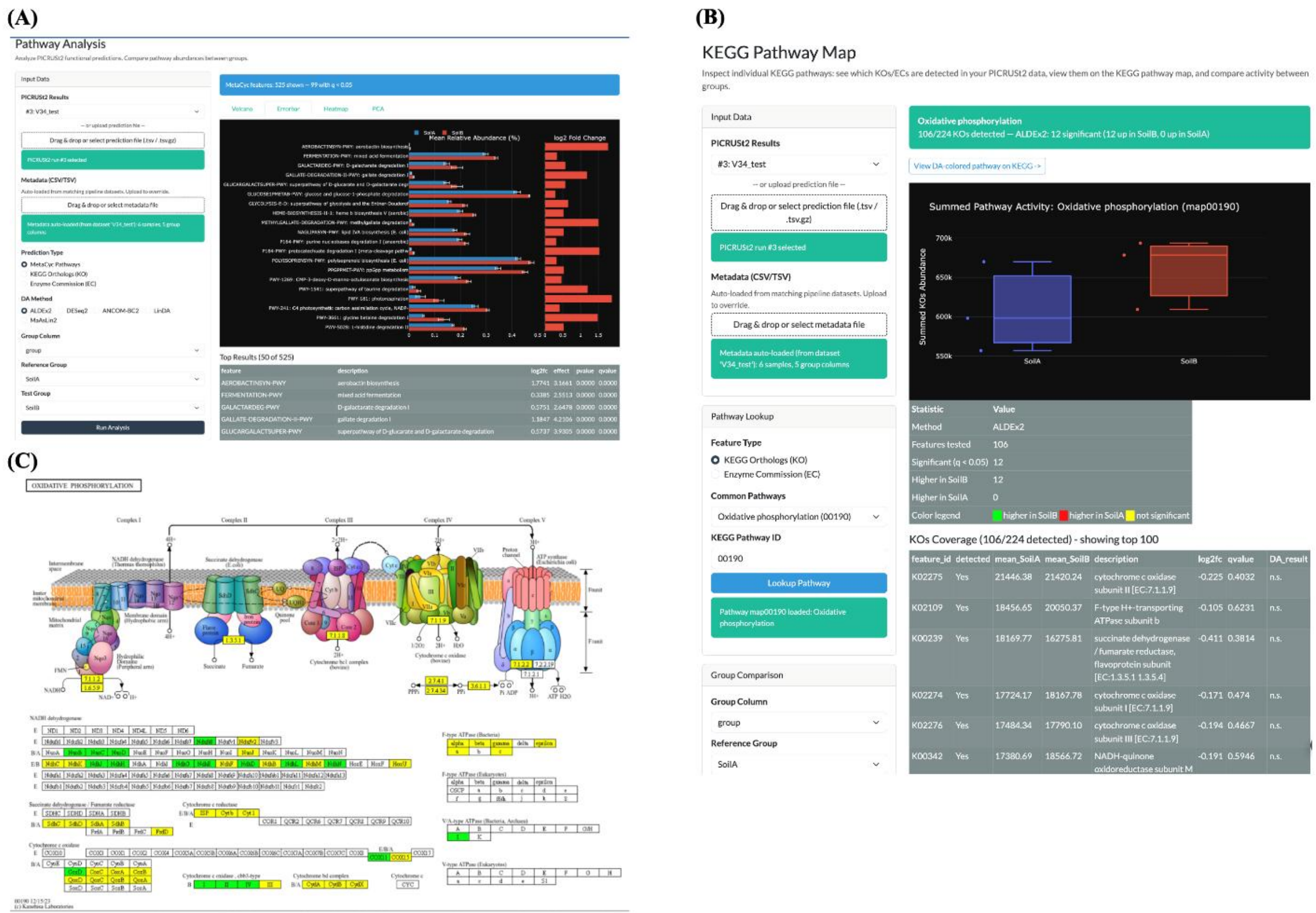

Fig. S1.

PICRUSt2 functional prediction and KEGG pathway analysis. (A) Pathway Analysis page showing MetaCyc pathway comparison between SoilA and SoilB with error bar plots and differential abundance results. (B) KEGG Pathway Map page for Oxidative phosphorylation (map00190), displaying KO coverage (106/224 KOs detected), pathway activity box plot comparing groups, and per-KO abundance table with statistical results. (C) KEGG pathway map with detected enzymes colored by differential abundance between groups.
