# Supplementary material for: 16S-Pipeline: A comprehensive web-based platform for end-to-end 16S rRNA amplicon sequencing analysis
Source: J Microbiol. 2026 May 14;64(5):e2603014. doi: 10.71150/jm.2603014 (PMC13577063; doi:10.71150/jm.2603014)
Supplement: Supplementary file 2 [file jm-2603014-Supplementary-Table-S1.pdf]

Table S1. Conda environments used by 16S-Pipeline

**Environment**	**Components**
microbiome_16S	Python 3.11, FastAPI, Dash, FastQC, Cutadapt, MAFFT, FastTree, vsearch, sra-tools
dada2_16S	R 4.3, DADA2 for denoising and taxonomy assignment
analysis_16S	R 4.4, ALDEx2, DESeq2, ANCOM-BC2
maaslin2_16S	R 4.3, MaAsLin2, LinDA, vegan
picrust2_16S	PICRUSt2 for functional prediction
