# Supplementary material for: 16S-Pipeline: A comprehensive web-based platform for end-to-end 16S rRNA amplicon sequencing analysis
Source: J Microbiol. 2026 May 14;64(5):e2603014. doi: 10.71150/jm.2603014 (PMC13577063; doi:10.71150/jm.2603014)
Supplement: Supplementary file 3 [file jm-2603014-Supplementary-Table-S2.pdf]

Table S2. Percentage of ASVs classified at each taxonomic rank across three sequencing platforms

Rank	V4 Illumina (%)	V3-V4 Illumina (%)	PacBio HiFi (%)
Kingdom	100.0	100.0	100.0
Phylum	99.5	99.6	99.9
Class	98.3	98.4	98.4
Order	88.4	86.2	91.3
Family	71.0	68.7	75.5
Genus	41.4	47.6	51.1
Species	0.0	0.0	0.8
