# Supplementary material for: 16S-Pipeline: A comprehensive web-based platform for end-to-end 16S rRNA amplicon sequencing analysis
Source: J Microbiol. 2026 May 14;64(5):e2603014. doi: 10.71150/jm.2603014 (PMC13577063; doi:10.71150/jm.2603014)
Supplement: Supplementary file 4 [file jm-2603014-Supplementary-Table-S3.pdf]

Table S3. DADA2 parameter settings used by 16S-Pipeline across Illumina paired-end, Illumina single-end, and long-read (PacBio HiFi/Nanopore) processing modes, indicating whether each parameter is fixed, automatically detected, or user-adjustable

Parameter	Illumina PE	Illumina SE	Long-read (PacBio/ONT)	Source
truncLen	Auto-detected or user-specified	Auto-detected or user-specified	Not applied (length filter instead)	User/auto
trimLeft	Auto-detected (leading N) or user-specified	Auto-detected or user-specified	Not applied	User/auto
maxN	0	0	0	Fixed
maxEE	c(5, 5)	5	10	Fixed (long-read user-adjustable)
truncQ	0 (disabled)	0 (disabled)	—	Fixed
rm.phix	TRUE	TRUE	FALSE	Fixed
errorEstimationFunction	loessErrfun	loessErrfun	PacBioErrfun (PacBio) / loessErrfun (Nanopore)	Fixed (platform-dependent)
BAND_SIZE	Default (16)	Default (16)	32	Fixed
HOMOPOLYMER_GAP_PENALTY	Default (NULL)	Default (NULL)	-1	Fixed
pool	FALSE (pseudo available)	FALSE (pseudo available)	FALSE (pseudo available)	User-adjustable
minOverlap	12	—	—	User-adjustable
maxMismatch	1	—	—	Fixed
minLen / maxLen	—	—	1000 / 1600	User-adjustable
Chimera removal method	consensus	consensus	consensus	Fixed
randomize (learnErrors)	TRUE	TRUE	TRUE	Fixed
